# Exploring the impact of stage and tumor site on colorectal cancer survival: Bayesian survival modeling

**DOI:** 10.1038/s41598-024-54943-8

**Published:** 2024-02-21

**Authors:** Shayesteh Alinia, Samira Ahmadi, Zahra Mohammadi, Farzaneh Rastkar Shirvandeh, Mohammad Asghari-Jafarabadi, Leila Mahmoudi, Malihe Safari, Ghodratollah Roshanaei

**Affiliations:** 1 Department of Statistics and Epidemiology, School of Medicine, Zanjan University of Medical Sciences, Mahdavi Blvd, Zanjan, 4513956111 Iran; 2 Cabrini Research, Cabrini Health, Malvern, VIC 3144 Australia; 3 School of Public Health and Preventative Medicine, Faculty of Medicine, Nursing and Health Sciences, Monash University, VIC, 3800 Australia; 4 Road Traffic Injury Research Center, Faculty of Health, Tabriz University of Medical Sciences, Golgasht St. Attar e Neshabouri St., Tabriz, 5166614711 Iran; 5 Department of Biostatistics, Medicine School, Arak University of Medical Sciences, Arak, Iran; 6 Modeling of Non-Communicable Diseases Research Canter, Department of Biostatistics, School of Public Health, Hamadan University of Medical Sciences, Hamadan, Iran

**Keywords:** Cancer site, Disease stage, Bayesian log-normal model, Semi-competing risks, Illness-death, Mortality, Cancer, Diseases, Gastroenterology, Medical research, Risk factors, Mathematics and computing

## Abstract

Colorectal cancer is a prevalent malignancy with global significance. This retrospective study aimed to investigate the influence of stage and tumor site on survival outcomes in 284 colorectal cancer patients diagnosed between 2001 and 2017. Patients were categorized into four groups based on tumor site (colon and rectum) and disease stage (early stage and advanced stage). Demographic characteristics, treatment modalities, and survival outcomes were recorded. Bayesian survival modeling was performed using semi-competing risks illness-death models with an accelerated failure time (AFT) approach, utilizing R 4.1 software. Results demonstrated significantly higher time ratios for disease recurrence (TR = 1.712, 95% CI 1.489–2.197), mortality without recurrence (TR = 1.933, 1.480–2.510), and mortality after recurrence (TR = 1.847, 1.147–2.178) in early-stage colon cancer compared to early-stage rectal cancer. Furthermore, patients with advanced-stage rectal cancer exhibited shorter survival times for disease recurrence than patients with early-stage colon cancer. The interaction effect between the disease site and cancer stage was not significant. These findings, derived from the optimal Bayesian log-normal model for terminal and non-terminal events, highlight the importance of early detection and effective management strategies for colon cancer. Early-stage colon cancer demonstrated improved survival rates for disease recurrence, mortality without recurrence, and mortality after recurrence compared to other stages. Early intervention and comprehensive care are crucial to enhance prognosis and minimize adverse events in colon cancer patients.

## Introduction

Colorectal cancer (CRC), also known as colon cancer or rectal cancer depending on its location, is a slowly progressing malignancy that originates as a tumor or abnormal tissue growth in the lining of the colon or rectum^1^. Although colon and rectal cancer are distinct entities, they are often collectively referred to as CRC due to their shared characteristics^2^. Globally, CRC ranks as the third most prevalent malignant neoplasm^3^ and the fourth leading cause of cancer-related mortality^4^. Among women, it is the third most common cancer, preceded by lung and breast cancer, while among men, it follows lung and prostate cancer^5,6^. The age-standardized incidence of colorectal cancer is reported as 19.7 per 100,000 individuals for both sexes, 23.6 for males, and 16.3 for females^5^. Furthermore, there is a concerning upward trend in the incidence of colon cancer worldwide, particularly in developing countries that have embraced a "Western" lifestyle^7^.

CRC is widely acknowledged as a multifactorial disease, with various factors such as diet, physical activity, genetics, and hormones playing a prominent role^8^. Moreover, certain lifestyle choices including obesity, sedentary behavior, and consumption of red meat, alcohol, and tobacco are considered to be contributory factors in the progression of colorectal cancer^7^. Consequently, mitigating the risk of developing this disease can be achieved by implementing measures that involve reducing and monitoring these factors, while concurrently increasing the intake of dietary fiber, wholesome foods, and specific vitamins^7,9–11^.

Cancer represents a paramount global public health and political challenge^12^. Over the past years, the economic impact and healthcare expenditures linked to CRC have experienced a steady increase. Notably, the cumulative healthcare costs for each CRC patient in China surpass the country's per capita gross domestic product (GDP) for the corresponding year^13,14^. Consequently, acquiring a comprehensive comprehension of this matter becomes indispensable.

The prognosis of CRC patients exhibits substantial variability, with 5-year survival rates ranging from 90 to 10%, contingent upon the stage of the disease and other pertinent factors^15^. Cancer staging, as outlined on the cancer.net website, entails precise determination of the cancer's location, identification of any metastasis, and evaluation of its impact on other bodily regions^16^. Recent studies have suggested that tumor localization and site possess some degree of association with clinical disease^17^ and it has also been indicated that variations in survival may arise due to disparities in biological characteristics and risk factors among different sites within the colon and rectum^18^. Numerous investigations have demonstrated noteworthy correlations between CRC patient survival and factors such as initial treatment modality, marital status, body mass index (BMI), tumor grade, and size^3,12,19^. Acquiring knowledge of the disease stage enables physicians to recommend the most appropriate therapeutic approach and aids in predicting the patient's prognosis, which refers to the likelihood of recovery^16^.

While there is a growing body of literature examining the prognostic significance of tumor location concerning overall survival, a further investigation involving large patient cohorts is still necessary^17^. Semi-competing risks provide flexible parametric and nonparametric specifications for survival functions within accelerated time-to-failure and proportional hazards models^20^. Moreover, the Bayesian paradigm offers a comprehensive framework for both estimation and predictive inference^21^. Our objective was to validate previously reported findings using our dataset of patients diagnosed with colorectal cancer. To accomplish this, we employed a recognized statistical approach, Bayesian survival modeling, along with a semi-competing risks model that accounted for both tumor site and disease stage (early and advanced).

## Methods

### Study design

**Figure 1:**
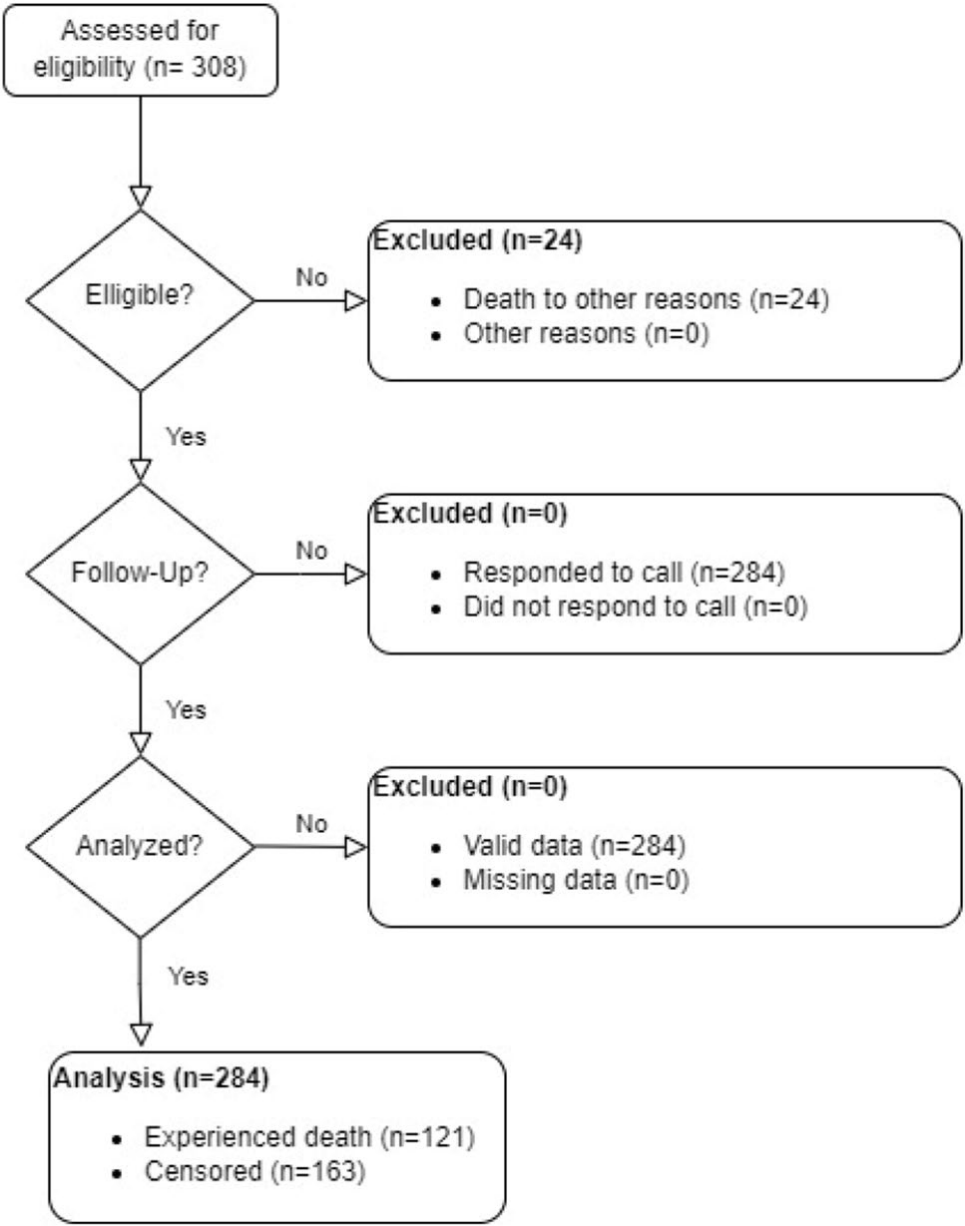
Flowchart of study steps.

This retrospective cohort study was conducted to investigate the impact of stage and tumor site on the survival outcomes of patients with colon cancer. The investigation included a total of 284 individuals who underwent surgical procedures at the Imam Khomeini (RA) clinic in Hamadan between 2001 and 2017. Comprehensive demographic and clinical information were extracted from the medical records of these patients. The primary objective of the study was to assess the relationship between stage, tumor site, and survival in individuals diagnosed with colorectal cancer. A total of 308 patients were enrolled in this study, out of which 284 were eligible for participation. Also, 131 patients experienced disease recurrence, and, 121 individuals died of the disease. The flowchart of the study steps is presented in Fig. 1.

### Study measurements

The patient files served as the primary data source for this study, providing comprehensive information on various demographic variables, including gender (categorized as male or female), age at diagnosis (expressed in years), and body mass index (BMI, measured in kg/m^2^). Additionally, clinical variables about surgery were extracted, encompassing radiotherapy, cancer site, chemotherapy, and morphology. These variables were categorized into binary groups (no: 0; yes: 1) and subjected to analysis. The grade, indicating the level of differentiation, was classified as good, moderate, or poor. The metastasis to other sites (no:0; yes:1), PT-stage(1:T2; 2:T3; 3:T4; 4:Tx), and PN-stage(1:N2; 2:N3; 3:N4; 4:Nx). The number of chemotherapy sessions was divided into three groups: individuals who did not undergo any chemotherapy sessions, those who attended 1 to 6 sessions, and those who attended more than 6 sessions. Tumor size was classified into three groups: 1: < 4, 2: >  = 4 < 7, and 3: >  = 7. Stage_site was divided into four groups, namely 1 (Early_RC), 2 (Early_CC), 3 (Adv_RC), and 4 (Adv_CC).

The patients for this study were selected based on their medical records. The inclusion criteria were patients who had undergone surgery for colorectal cancer. The exclusion criteria were patients who had other types of cancer or who had not undergone surgery. The rationale behind these choices was to focus on the recurrence of colorectal cancer post-surgery. To calculate the time until recurrence, we used the date of surgery as the starting point and the date of local or distant recurrence as the endpoint. The time was calculated in months. Patients who did not have a recurrence until the end of the study were considered censored for the occurrence of recurrence. Regarding the vital status and date of death, this information was obtained through medical and administrative record sources. In this study, all deaths were considered colorectal cancer-related death. In cases where patients passed away during the study period, they were considered censored observations, and their contact information was recorded for subsequent telephone follow-up to update their status in the patient files.

### Study size

In the present study, the sufficiency of the sample size was evaluated by considering the number of variables under investigation. It is commonly advised to have a minimum of 15 samples per observed variable to ensure statistical robustness. Therefore, given the presence of 13 identified risk factors, the inclusion of 284 patients in our study is deemed satisfactory for analysis^22^.

### Statistical analysis

Within this research, we utilized various statistical methods and modeling techniques to analyze and present the data. Specifically, for numerical variables, such as mean and median, we employed descriptive statistics to showcase central tendencies and variability, using standard deviation (SD) and minimum–maximum (min–max) for dispersion. For categorical variables, we reported prevalence as percentages. Significant variables identified in the multivariable analysis were used to plot adjusted survival curves. To compare probabilities between groups for each semi-competing risk, we employed a cause-specific log-rank test. The groups under investigation were colon and rectal cancer patients, categorized by disease stage (early stage and advanced stage). The inclusion of variables in the multivariate analysis was based on their significance level (p < 0.1) in the univariate analysis.

To assess the relationship between covariate variables and outcomes, such as the probability of recurrence, death without recurrence, and death after experiencing recurrence, we employed a semi-competitive analysis of Bayesian survival models under the accelerated failure time to death model (AFT). The analysis involved a Markov chain Monte Carlo (MCMC) random sampling algorithm for generating samples. Additionally, we explored the interaction between disease stage and cancer location as predictors of recurrence and death without recurrence using an independent Bayesian AFT model with a log-normal survival distribution. In cases where the interaction between these two variables was not significant, we ran the model without the interaction.

For model evaluation, we considered the deviance information criterion (DIC)^23^ and the logarithm of the pseudo marginal likelihood (LPML)^24^ as measures. The model with interaction between variables outperformed the model without interaction based on these evaluation metrics. To assess differences in survival times between different groups of covariates, we employed Kaplan–Meier plots. However, these plots were inadequate to illustrate the disparities in survival and recurrence between colon and rectal cancer patients concerning covariate variables. To address this limitation, we utilized the log-rank test^25^. All the analyses and modeling were implemented using R 4.1 software (https://www.rproject.org/).

### Bayesian survival analysis with log-normal model

Bayesian approaches are favored over frequentist approaches in survival analysis due to their superior information power, combining probabilistic data with prior knowledge of parameter distributions. Parametric models play a crucial role in Bayesian survival analysis, as they form the foundation for much of the actual Bayesian analysis. Among the popular parametric survival models, the log-normal model stands out^26^. The log-normal distribution is particularly relevant when the cause of death or failure results from the accumulation of additive damages over time^27^. In this study, the log-normal method is employed, assuming that the logarithms of survival times follow a normal distribution The MCMC method is utilized to generate samples from the posterior density, enabling the approximation of expectations for quantities of interest in this study^24^.

### Semi-competing risks

Semi-competing risks refer to situations in time-to-event analysis where the occurrence of a non-terminal event depends on the prior occurrence of a terminal event, but not vice versa. Employing models that account for semi-competing risks enables the investigation of associations between covariates and the simultaneous timing of outcomes^28^. The data involving semi-competing risks can be effectively modeled using the illness-death approach with joint frailty. However, the estimation process often relies on the subjective specification of the parametric frailty distribution^29^.

### Bayesian AFT models for independent semi-competing risks

To compare the survival times, the AFT model was employed, assuming that the effects of covariates on the survival time are multiplicative^30^.

The AFT model is represented by the following equations:

1 $$log({T}_{i1})= {X}_{i1}^{T}{\beta }_{1}+ {\gamma }_{i}+{\varepsilon }_{i1}, {T}_{i1}>0$$

2 $$log({T}_{i2})= {X}_{i2}^{T}{\beta }_{2}+ {\gamma }_{i}+{\varepsilon }_{i2}, {T}_{i2}>0$$

3 $$log \left({T}_{i2}- {T}_{i1}\right) = {X}_{i3}^{T}{\beta }_{3}+ {\gamma }_{i}+{\varepsilon }_{i3}, {T}_{i1}>{T}_{i2}$$

According to the mentioned formulas, Eqs. (1), (2) and (3) refer to the probability of recurrence of CRC, the probability of death from CRC without any recurrence, and the probability of death from CRC after any recurrence, respectively. ${x}_{ig}$ signifies the vector of transition-specific covariates, and ${B}_{ig}$ represents the vector of transition-specific regression parameters.

In each of the expressions (1), (2) and (3), ${\gamma }_{i}$ presents a study subject-specific frailty, which introduces positive dependence between recurrence and death without recurrence. It is assumed that ${\gamma }_{i}$ follows a normal distribution with a mean of zero and a variance of θ. The variance component θ is assumed to follow a conjugate inverse gamma distribution, denoted as IG(a(θ), b(θ)).

Furthermore, the non-informative flat priors are assumed for the log-normal errors parameters subscription $({\mu }_{g})$ and the denoted for subscription errors $({\varepsilon }_{ig})$. For ${\mu }_{g}$ non − informative flat priors on the real line are considered, while independent inverse gamma distributions, denote as IG (${a}_{g}^{(\sigma )}$, ${b}_{g}^{(\sigma )}$, are used for ${\sigma }_{g}^{2}$.

### Ethics approval and consent to participate

The institutional review board of Zanjan University of Medical Sciences approved the protocol of the study (ethics code: IR.ZUMS.REC.1400.419). The participants' privacy was preserved. All participants filled out and signed the informed consent and assent. Also, all methods were carried out according to relevant guidelines and regulations.

## Result

### Profile of the patients

In this scientific investigation involving a cohort of 284 patients, we conducted an extensive analysis to investigate the occurrence of disease recurrence and mortality. Out of the total patient population, 131 individuals (46.1%) experienced disease recurrence, while 121 individuals (42.6%) succumbed to the disease. The gender distribution of the patients revealed that among the total cohort, 134 individuals (47.2%) were women, and among those who died, 71 individuals (52.9%) were men. Furthermore, out of the patients who experienced disease recurrence, 56 individuals (42.7%) were women.

The patients were stratified into three age groups based on their age at diagnosis: less than 50 years, 51 to 70 years, and more than 70 years. Notably, the second age category constituted the largest subgroup, comprising 158 patients (55.6%). Within this subgroup, there were 65 deaths (53.7%) and 72 cases of disease recurrence (55.0%). As for the patients' body weight, it was observed that overweight individuals accounted for 180 patients (63.4%). Among this group, there were 72 deaths (59.5%) and 80 cases of disease recurrence (61.1%).

Chemotherapy was administered to the majority of patients, comprising 85.6% of the total study population. Among these individuals, 119 patients (90.8%) experienced disease recurrence, and 109 patients (90.1%) succumbed to the disease. On the other hand, 41 patients (14.4%) did not receive chemotherapy, with 12 cases (9.2%) exhibiting disease recurrence and 12 cases (9.9%) resulting in mortality. Furthermore, more than six sessions of chemotherapy were administered to 148 patients (52.1%), among whom 76 individuals (58.0%) experienced disease recurrence, while 61 individuals (50.4%) passed away.

The patient population was further categorized based on the stage of cancer. A total of 76 individuals (26.8%) were classified as level Early_RC (patients with early-stage rectal cancer), while 141 individuals (49.6%) were classified as level Early_CC (patients with early-stage colon cancer). Moreover, 23 individuals (8.1%) were classified as level Adv_RC (patients with advanced-stage rectal cancer), and 44 individuals (15.5%) were classified as level Adv_CC (patients with advanced-stage colon cancer). The median survival time was estimated to be 61.0 months, with a 95% confidence interval ranging from 42.2 to 79.8 months. Additionally, the study calculated the probabilities of survival at 1 year, 3 years, and 5 years, along with their corresponding 95% confidence intervals. These probabilities were determined to be 86.9%, 62.1%, and 50.4%, respectively (Table 1).

**Table 1:** Participants’ profile.

Characteristics	Frequency	Percentage
Age at diagnosis (years)		
$\le$ 50	91	32
51–70	158	55.6
$\ge$ 70	35	12.3
Gender		
Female	134	47.2
Male	150	52.8
Grade (differentiation level)		
Well-differentiated	117	41.2
Moderately differentiated	145	51.1
Poorly differentiated	22	7.7
BMI		
Normal	46	16.2
Overweight	180	63.4
Obese	58	20.4
Cancer site		
Colon	185	65.1
Rectum	99	34.9
Radiotherapy(yes)	89	31.3
Chemotherapy(yes)	243	85.6
Morphology(adeno)	281	98.9
Tumor size		
< 4	72	25.4
$\ge$ 4 < 7	160	56.3
$\ge$ 7	52	18.3
Stage site		
Rc—early stage	76	26.8
Cc—early stage	141	49.6
Rc—advanced stage	23	8.1
Cc—advanced stage	44	15.5

*BMI* body mass index, *RC* rectal cancer, *CC* colon Cancer.

### Result of log-rank tests

**Figure 2:**
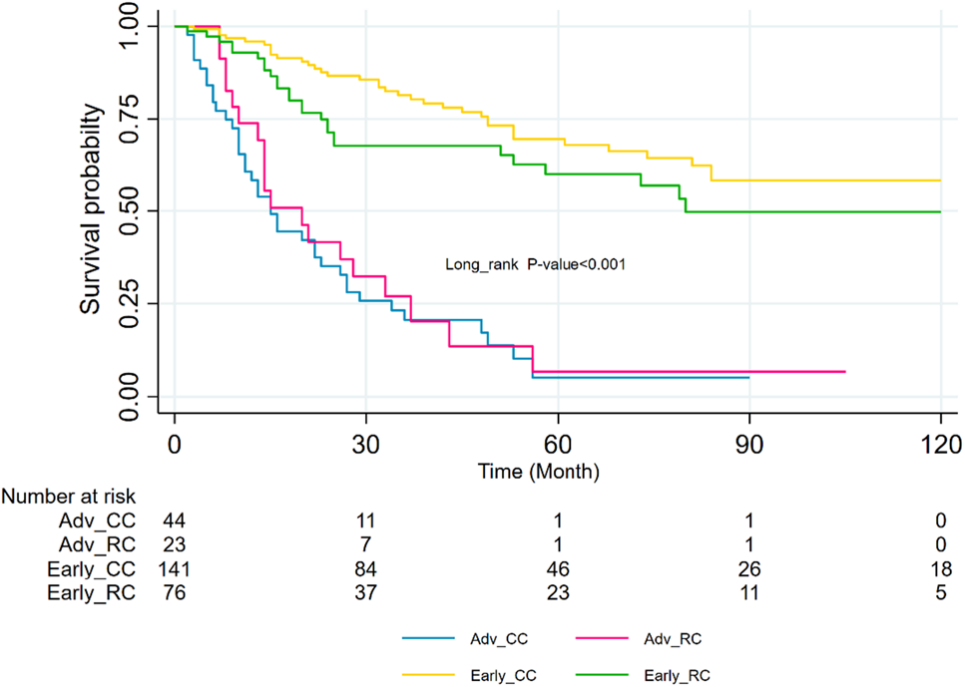
Kaplan–Meier survival curves by stage of the cancer for colon and rectal cancer patients for survival. *Early_RC* Early stage, rectal cancer, *Early_CC* Early stage, colon cancer, *Adv_CC* Advanced stage, colon cancer, *Adv_RC* Advanced stage, rectal cancer.

**Figure 3:**
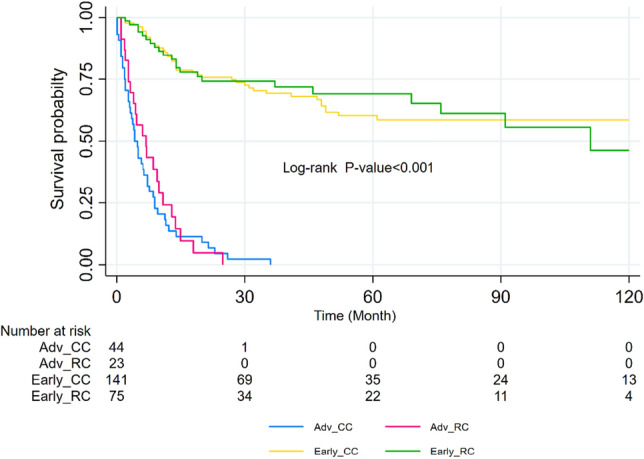
Kaplan–Meier survival curves by stage of the cancer for colon and rectal cancer patients for recurrence. *Early_RC* Early stage, rectal cancer, *Early_CC* Early stage, colon cancer, *Adv_CC* Advanced stage, colon cancer, *Adv_RC* Advanced stage, rectal cancer.

The log-rank test demonstrated a statistically significant association between age at diagnosis and survival (P-value < 0.001), indicating that patients over the age of 70 exhibited lower survival rates. Furthermore, higher age categories were significantly correlated with increased rates of recurrence outcomes. Additionally, disease stage was identified as a significant predictor for both terminal and non-terminal events (P-value < 0.001), with more advanced stages showing a significant increase in the rate of both types of events. Patients with advanced disease stages experienced lower survival rates for all three events. Furthermore, patients who received fewer than six chemotherapy sessions exhibited a higher rate of events compared to those who did not receive any chemotherapy. Nevertheless, when the number of chemotherapy sessions exceeded six, the rate of events decreased (P-value = 0.057). The study also examined four composite groups based on the stage of the disease and site, colon and rectum. The log-rank test indicated a significant difference between the groups in terms of mortality outcome (P-value < 0.001) (Fig. 2). Also, for the recurrence outcome, the results revealed that patients in the Early_RC and Early_CC groups had better survival rates, while those in the Adv_RC and Adv_CC had lower survival rates (Fig. 3). Additionally, patients in the early stage of the disease in both groups had lower recurrence rates, whereas those in the advanced stage had higher recurrence rates (Fig. 3). Moreover, when comparing the occurrence rate between non-terminal and terminal events, it became evident that the rate was considerably higher in the recurrence outcome compared to the death outcome.

### Result of Bayesian AFT log-normal model

According to Table 2, the findings demonstrated that as age at diagnosis increased, there was an associated increase in the survival time ratio for recurrence (Time Ratio = 1.797; 95% Confidence Interval [CI] 1.514–2.199), death without recurrence (TR = 1.919; 95% CI 1.755—2.056), and death after recurrence (TR = 1.034; 95% CI 0.964–1.289). In male patients, a significantly higher time ratio of death without recurrence (TR = 1.522; 95% CI 1.017–1.632) and death after recurrence (TR = 1.632; 95% CI 1.188–2.473) was observed compared to female patients, but no significant impact on recurrence was noted. Additionally, an increase in the number of chemotherapy sessions was significantly associated with longer survival times in all three cases: recurrence (TR = 1.690; 95% CI 1.490–1.855), death without recurrence (TR = 2.113; 95% CI 1.481–2.929), and death after recurrence (TR = 1.694; 95% CI 1.271–1.986). This indicated that a higher number of chemotherapy sessions resulted in a greater ratio of survival time in each case.

**Table 2:** Predictors of nonterminal and terminal events utilizing Bayesian Independent AFT model with log-Normal baseline survival distribution for interaction between Disease stage and cancer site.

		Recurrence		Death without recurrence		Death after recurrence	
		TR	95% CI	TR	95% CI	TR	95% CI
Age at diagnosis (years)	Trend effect	1.797	1.514–2.199*	1.919	1.755–2.056*	1.034	0.964–1.289*
Gender	Male	1.211	0.888–1.739	1.522	1.017–1.632*	1.632	1.188–2.473*
Number of chemotherapies	Trend effect	1.690	1.490–1.855*	2.113	1.481–2.929*	1.694	1.271–1.986*
Grade (differentiation level)	Well	Referent	–	–	–	–	–
	Moderate	1.965	1.338–2.349*	2.612	1.821 _3.648*	1.093	0.932–1.225
	Poor	1.041	0.619–1.870	2.076	1.687–2.443*	0.716	0.388–0.984*
Tumor size	Trend effect	NC	NC	1.574	1.259–2.148*	1.008	0.740–1.094
Disease stage	Trend effect	0.877	0.625–1.365	2.188	1.941–3.437	1.223	1.067–1.613*
Cancer site	Trend effect	1.249	1.147–1.416*	1.322	1.071–1.845*	0.742	0.603–1.132
Disease stage # Cancer site	Trend effect	1.432	0.950–2.170	1.149	0.553–1.390	0.963	0.808–1.405

Deviance information criterion (DIC = 1866), Logarithm of the pseudo marginal likelihood (LPML = − 882).

The frailty component was significant in the multivariable model (Variance of frailties: 0.775, 95% CI (0.684–0.974)).

Trend effect: The model considered the trend effect for ordinal categorical variables. The variables BMI category, surgery, radiotherapy, chemotherapy, morphology could not be entered in the model in the multivariable model (All P > 0.05).

*NC* not computable, *CI* credibility interval, *TR* time ratio.

*P < 0.05.

When comparing well-differentiated tumors to those with moderate differentiation, tumors with moderate differentiation displayed a higher time ratio for recurrence (TR = 1.965; 95% CI 1.338–2.349) and death without recurrence (TR = 2.612; 95% CI 1.821–3.648). Conversely, tumors with a poor level of differentiation were significantly associated with higher rates and a greater time ratio of death without recurrence (TR = 2.076; 95% CI 1.687–2.443) and a lower time ratio of death after recurrence (TR = 0.716; 95% CI 0.388–0.984). Furthermore, a larger tumor size demonstrated a significant association with higher rates of death after recurrence and an increased time ratio (TR = 1.574; 95% CI 1.259–2.148). The disease stage also influenced the time ratio of death after relapse (TR = 1.223; 95% CI 1.067–1.613), as a more advanced stage was significantly associated with higher rates of death after recurrence but did not exhibit a significant effect on recurrence and death without recurrence. Regarding the cancer site, the survival time ratio for recurrence (TR = 1.249; 95% CI 1.147–1.416) and death without recurrence (TR = 1.322; 95% CI 1.071–1.845) was found to be higher. However, the interaction between disease stage and cancer site did not yield significant effects on any of the outcomes.

According to the findings presented in Table 3, increasing age was associated with a higher time ratio for recurrence (TR = 1.478; 95% CI 1.293–2.080), death without recurrence (TR = 2.210; 95% CI 1.676–2.792), and death after recurrence (TR = 1.272; 95% CI 1.163–1.443). In comparison to females, males exhibited a significantly higher time ratio for recurrence (TR = 1.316; 95% CI 1.055–1.518), death without recurrence (TR = 2.420; 95% CI 1.741–3.658), and death after recurrence (TR = 1.535; 95% CI 1.063–1.807).

**Table 3:** Predictors of nonterminal and terminal events utilizing Bayesian Independent AFT model with log-Normal baseline survival distribution.

		Recurrence		Death without recurrence		Death after recurrence	
		TR	95% CI	TR	95% CI	TR	95% CI
Age at diagnosis (years)	Trend effect	1.478	1.293–2.080*	2.210	1.676–2.792*	1.272	1.163–1.443*
Gender	Male	1.316	1.055–1.518*	2.420	1.741–3.658*	1.535	1.063–1.807*
Number of chemotherapies	Trend effect	1.533	1.163–1.896*	1.616	1.312–2.051*	1.623	1.382–2.254*
Grade (differentiation level)	Well	Referent	–	–	–	–	–
	Moderate	1.368	1.057–1.490*	2.391	1.836–3.174*	1.100	0.822–1.259
	Poor	1.587	0.803–1.785	1.580	1.164–2.957*	0.922	0.697–1.187
Tumor size	Trend effect	1.259	1.143–1.522*	2.435	1.824–2.848*	1.118	0.854–1.528
Stage—site	Early_RC	Referent	–	–	–	–	–
	Early_CC	1.712	1.489–2.197*	1.933	1.480–2.510*	1.847	1.147–2.178*
	Adv_RC	0.665	0.484–0.721*	1.399	0.829–1.572	1.203	0.933–1.478
	Adv_CC	0.847	0.446–0.919	2.743	1.569–4.344	1.065	0.810–1.474

Deviance information criterion (DIC = 1927), Logarithm of the pseudo marginal likelihood (LPML =  − 821).

The frailty component was significant in the multivariable model (Variance of frailties: 0.719, 95% CI (0.595–0.875)).

Trend effect: The model considered the trend effect for ordinal categorical variables. The variables BMI category, surgery, radiotherapy, chemotherapy, morphology could not be entered in the model in the multivariable model (All P > 0.05).

Early_RC: Patients with early stage, rectal cancer.

Early_CC: Patients with early stage, colon cancer.

Adv_CC: Patients with advanced stage, colon cancer.

Adv_RC: Patients with advanced stage, rectal cancer.

*CI* credibility interval, *TR* time ratio.

*P < 0.05.

An increase in the number of chemotherapy sessions was associated with a higher time ratio for recurrence (TR = 1.533; 95% CI 1.163–1.896), death without recurrence (TR = 1.616; 95% CI 1.312–2.051), and death after recurrence (TR = 1.623; 95% CI 1.382–2.254). When well-differentiated tumors were compared to those with a moderate level of differentiation, tumors with moderate differentiation showed a higher time ratio for recurrence (TR = 1.368; 95% CI 1.057–1.490) and death without recurrence (TR = 2.391; 95% CI 1.836–3.174). Similarly, tumors with a poor level of differentiation had a higher time ratio for death without recurrence (TR = 1.580; 95% CI 1.164–2.957). Additionally, larger tumor size was associated with a higher time ratio for recurrence (TR = 1.259; 95% CI 1.143–1.522) and death without recurrence (TR = 2.435; 95% CI 1.824–2.848).

When patients with Early_RC were compared to Early_CC, patients with early-stage colon cancer exhibited a higher time ratio for recurrence (TR = 1.712; 95% CI 1.489–2.197), death without recurrence (TR = 1.933; 95% CI 1.480–2.510), and death after recurrence (TR = 1.847; 95% CI 1.147–2.178). Conversely, patients with Adv_RC showed a lower time ratio for recurrence (TR = 0.665; 95% CI 0.484–0.721).

## Discussion

The objective of this study was to examine the occurrence of non-terminal events (recurrence), the likelihood of terminal events (death without recurrence), and the conditional probability of terminal events given non-terminal events (death after recurrence) in cancer patients. The Bayesian AFT Log-Normal method was employed to analyze the effects of disease location and stage on these outcomes. A total of 284 patients participated in this study, and the findings indicated a recurrence rate of 46.1% and a mortality rate of 42.6%. Gender was found to exert an impact on these outcomes, with 58.7% of deceased patients being male and 42.7% of patients experiencing recurrence being female. Regarding the comparison between colorectal and rectal cancer groups, the results revealed that early-stage groups exhibited higher rates of survival and recurrence, while advanced-stage groups demonstrated lower survival rates. Moreover, the recurrence rate surpassed the mortality rate. The Bayesian AFT Log-Normal model outcomes further highlighted the significance of age, the number of chemotherapy sessions, and tumor size in influencing survival rates and durations across different scenarios. Additionally, gender and cancer stage were identified as notable factors influencing survival time.

Researchers commonly employ the illness-death model in their studies due to its association with widely used survival analysis techniques and its accessibility through available software. Nevertheless, it is crucial to acknowledge that the hazard ratio, which is commonly utilized as a measure in survival analysis, is not the sole metric available for calculating and reporting outcomes. An alternative approach that provides a comprehensive and straightforward explanation is the accelerated failure time (AFT) model. This model facilitates the modeling of the logarithm of survival time, taking into account the explanatory variables^31^.

Bayesian analysis endeavors to strike a harmonious equilibrium between prior knowledge and empirical evidence derived from data. While strong prior beliefs can support weak evidence arising from limited data, it is crucial to avoid overshadowing the actual data. Sensitivity analysis is performed to investigate the influence of different prior choices, which is often a contentious aspect. Non-informative priors, which assign equal probabilities to all possible parameter values, offer a potential solution to achieve balance and mitigate subjectivity in Bayesian analysis. Notably, even when employing improper priors, Bayesian analysis can produce proper posterior distributions^32^. The Bayesian approach presents a practical and valuable alternative to frequentist methods, particularly aided by advancements in computational techniques and the availability of software tools. These factors contribute to the feasibility and relevance of the proposed AFT illness-death model, which serves as a valuable complement to conventional hazard-based approaches^33^.

The clinical relevance of our findings lies in the potential impact on patient care and treatment strategies. The significant factors identified in our study, such as age, the number of chemotherapy sessions, tumor size, gender, and cancer stage, could inform personalized treatment plans and prognostic discussions. Moreover, understanding the recurrence and survival patterns in early and advanced-stage groups could guide surveillance strategies post-treatment.

The findings of this investigation are in line with previous research conducted on predicting post-surgery patient survival in colon cancer^3,30,34^. Siân A Pugh et al. (2016) conducted a study demonstrating that the probability and site of recurrence, as well as overall survival, are influenced by the location and stage of the primary tumor^35^. Similarly, Ryuk JP et al. (2014) revealed in their study that the occurrence of local recurrence in patients with colon cancer is lower compared to patients with rectal cancer. These results align with our findings, highlighting the impact of primary tumor location and disease stage on recurrence and survival^36^. The influence of recurrence on survival and mortality within the first five years after curative resection has been documented in several studies^37–39^. Recent studies have also reported similar findings. For instance, a study published in 2021 reported that patients with stage I disease and T1- and N0-tumor had the highest probability of cure (94%, 95% and 90%, respectively), while those with a T4-tumor or N2-tumor had the lowest probability of cure (62% and 50%, respectively)^40^. Another study in 2022 reported that 1, 3, and 5 years’ survival rates were 90, 70, and 63% for all the patients, 89%, 67%, and 58% for rectal cancer and 90%, 74%, and 71% for colon cancer, respectively^41^.

The results obtained from a study conducted in 2020, which aimed to investigate the differences in survival rates for colon and rectal cancer based on age and stage of diagnosis in seven high-income countries with health systems, showed that there are differences in 1-year and 5-year net survival rates for colon and rectal cancer according to the stage of diagnosis, age, and country. Additionally, differences in the stage distribution of colon and rectal cancer based on age were observed in different countries, with clear differences in the survival rates of patients with metastatic disease and those diagnosed at an older age (regardless of the stage). The results of this study were consistent with the findings of the present study^42^.

In our study, we found similar trends in colon cancer status across four European countries, considering factors such as age, gender, and disease stage. All patients aged 18 to 99 years diagnosed with primary, invasive colorectal adenocarcinoma were included. Notably, England saw the most significant increase in surgical resection for stage III rectal cancer patients. Survival rates for stage I patients were consistent across all countries, while England had lower 3-year survival rates for stage II or III rectal cancer and stage IV colon cancer. In Sweden, a wider survival range was observed for rectal cancer patients older than 75 years, indicating the impact of the disease stage on survival, aligning with our findings^43^.

Consistent with the present study, Baghestani demonstrated a significant association between age at diagnosis and patient survival time^44^, which is in line with findings reported in other studies^45,46^.

## Strengths and limitations

While the study offers valuable insights, it is important to acknowledge and consider certain limitations. Firstly, the retrospective nature of the study introduces potential biases and confounding variables as the data was collected from patient records after the events occurred, rather than through a controlled experiment. This retrospective design may limit the ability to establish causal relationships or account for all relevant factors. Secondly, the study was conducted at a single center, which restricts the generalizability of the findings to other populations. Variations in patient demographics, healthcare practices, and treatment protocols across different centers and regions might influence the applicability of the results on a broader scale. Additionally, the study did not account for certain crucial factors that could impact the prognosis of colon cancer, including comorbidities, genetic factors, and lifestyle variables. The omission of these important variables might limit the comprehensiveness and accuracy of the findings, as these factors have been recognized to play significant roles in the prognosis and outcomes of colon cancer patients.

On the other hand, this study offers significant contributions to our understanding of colorectal cancer patient survival and boasts several notable strengths. Firstly, the inclusion of a relatively large sample size comprising 284 patients ensures a high level of confidence in the findings. By utilizing Bayesian methodology for survival modeling, the study permits the examination of various factors and yields probabilistic outcomes. Moreover, the research encompasses a comprehensive analysis of the impact of disease stages and tumor location on survival outcomes among colorectal cancer patients. The adoption of the log-rank test and the Bayesian accelerated failure time model further enhances the robustness and reliability of the results, particularly in the context of well-differentiated tumors and larger tumor sizes.

## Conclusion

In summary, this study employed a Bayesian approach to survival modeling to investigate the impact of stage and tumor site on the survival of colorectal cancer patients. The results highlight the significant influence of both stage and tumor sites on survival outcomes. Specifically, patients with early-stage colon cancer exhibited higher rates of survival for disease recurrence, mortality without recurrence, and mortality after recurrence compared to patients in other stages. These findings underscore the critical importance of early detection and effective management strategies for colon cancer patients, as they can significantly improve prognosis and mitigate the risk of adverse events. Furthermore, age at diagnosis, the number of chemotherapy sessions, tumor differentiation level, and tumor size displayed significant associations with survival outcomes. These factors contribute to the overall prognosis and treatment response of colorectal cancer patients. Future investigations should consider exploring the combined effects of additional factors, such as environmental risk factors, overall health status, and family history, to gain a comprehensive understanding of their impact on survival. Additionally, long-term follow-up studies and validation of the current findings in larger patient populations would provide valuable insights into the enduring impact of stage and tumor site on survival in colorectal cancer patients.

Future direction could be first, utilizing survival prediction models; the study found that age, the number of chemotherapy sessions, tumor size, gender, and cancer stage significantly influence survival rates and durations. These factors can be incorporated into survival prediction models, such as machine learning methods and neural networks, to enhance their accuracy and predictive capability. Second, consideration of additional influential factors; the study highlighted the impact of certain factors on survival outcomes, which limits the results for clinical decision-making. Examining additional factors can provide a more comprehensive understanding of their influence on survival, which aligns with the study’s findings. Factors such as patient age, specific treatment modalities received, overall health status, and other relevant variables including comorbidities, clinical, and pathological should be considered to provide a more comprehensive understanding of their influence on survival. Finally, practical application of findings: The study’s findings can be translated into clinical practice to improve the conditions and treatment of colorectal cancer patients according to the detailed finding of this study focusing on the stage and site of the cancer. By incorporating these suggestions into future studies, researchers can advance their understanding of the impact of disease stages and tumor location on survival in colorectal cancer patients, leading to improved prognostic and therapeutic approaches in clinical practice.

## Data Availability

The data that support the findings of this study are not publicly available. Data are, however, available from the authors upon reasonable request by MAJ.
